# Using Monozygotic Twins to Dissect Common Genes in Posttraumatic Stress Disorder and Migraine

**DOI:** 10.3389/fnins.2021.678350

**Published:** 2021-06-22

**Authors:** Charlotte K. Bainomugisa, Heidi G. Sutherland, Richard Parker, Allan F. Mcrae, Larisa M. Haupt, Lyn R. Griffiths, Andrew Heath, Elliot C. Nelson, Margaret J. Wright, Ian B. Hickie, Nicholas G. Martin, Dale R. Nyholt, Divya Mehta

**Affiliations:** ^1^Centre for Genomics and Personalised Health, School of Biomedical Science, Faculty of Health, Queensland University of Technology, Kelvin Grove, QLD, Australia; ^2^Centre for Genomics and Personalised Health, Genomics Research Centre, School of Biomedical Sciences, Institute of Health and Biomedical Innovation, Kelvin Grove, QLD, Australia; ^3^QIMR Berghofer Medical Research Institute, Royal Brisbane Hospital, Herston, QLD, Australia; ^4^Institute for Molecular Bioscience, The University of Queensland, Brisbane, QLD, Australia; ^5^Department of Psychiatry, Washington University School of Medicine in St. Louis, St. Louis, MO, United States; ^6^Queensland Brain Institute, The University of Queensland, Brisbane, QLD, Australia; ^7^Centre for Advanced Imaging, The University of Queensland, Brisbane, QLD, Australia; ^8^Brain and Mind Centre, The University of Sydney, Sydney, NSW, Australia

**Keywords:** DNA methylation, twins, posttraumatic stress disorder, migraine, genes

## Abstract

Epigenetic mechanisms have been associated with genes involved in Posttraumatic stress disorder (PTSD). PTSD often co-occurs with other health conditions such as depression, cardiovascular disorder and respiratory illnesses. PTSD and migraine have previously been reported to be symptomatically positively correlated with each other, but little is known about the genes involved. The aim of this study was to understand the comorbidity between PTSD and migraine using a monozygotic twin disease discordant study design in six pairs of monozygotic twins discordant for PTSD and 15 pairs of monozygotic twins discordant for migraine. DNA from peripheral blood was run on Illumina EPIC arrays and analyzed. Multiple testing correction was performed using the Bonferroni method and 10% false discovery rate (FDR). We validated 11 candidate genes previously associated with PTSD including *DOCK2*, *DICER1*, and *ADCYAP*1. In the epigenome-wide scan, seven novel CpGs were significantly associated with PTSD within/near *IL37*, *WNT3*, *ADNP2*, *HTT*, *SLFN11*, and *NQO2*, with all CpGs except the *IL37* CpG hypermethylated in PTSD. These results were significantly enriched for genes whose DNA methylation was previously associated with migraine (*p*-value = 0.036). At 10% FDR, 132 CpGs in 99 genes associated with PTSD were also associated with migraine in the migraine twin samples. Genes associated with PTSD were overrepresented in vascular smooth muscle, axon guidance and oxytocin signaling pathways, while genes associated with both PTSD and migraine were enriched for AMPK signaling and longevity regulating pathways. In conclusion, these results suggest that common genes and pathways are likely involved in PTSD and migraine, explaining at least in part the co-morbidity between the two disorders.

## Introduction

Post-traumatic stress disorder (PTSD) is a debilitating, stress-related psychiatric condition, which occurs among persons exposed to traumatic events involving life threats, serious injury, or death (Matosin et al., 2017). It develops as a result of failure to contain the normal stress response hence dysregulation of the hypothalamic–pituitary–adrenal (HPA) axis which is one of the body’s major stress response systems (Wong, 2002; Yehuda and LeDoux, 2007). Although nearly 90% of individuals are exposed to a traumatic event during their lifetime (Breslau et al., 1998), only a small proportion go on to develop PTSD (Matosin et al., 2017). There is significant variation in the risk of PTSD among individuals experiencing the same trauma; this is likely determined by genetic predisposition and epigenetic mechanisms (Kessler et al., 2017). One of the best understood epigenetic mechanisms is DNA methylation through the addition of a methyl chemical bond to the cytosine C5 in the cytosine-phosphate-guanine (CpG) dinucleotides of the DNA, influencing gene activity and considered to be a stable epigenetic mark in post-mitotic cells (Guo et al., 2011). Generally, DNA methylation, especially if occurring close to the promoter region of the gene is known to inhibit gene transcription.

Epigenome-wide Association Studies (EWAS) provide an unbiased approach to identify DNA methylation loci associated with a disease independently of what is known about the pathophysiology (Almli et al., 2014). Most EWAS examining epigenetic effects of trauma exposure use DNA methylation profiles in peripheral tissues like blood and saliva, which are useful for biomarker identification (Almli et al., 2014). Several genes previously associated with stress or epigenetic regulation of neuronal function have been identified through EWAS of PTSD (Melroy-Greif et al., 2017; Nievergelt et al., 2018; Howie et al., 2019). Additionally, multiple genome-wide studies have also uncovered genetic contributions to PTSD risk and symptomatology through candidate genes which have encoded for varied proteins (Gormley et al., 2016; Kilaru et al., 2016; Blacker et al., 2019). EWAS show that there are significant epigenetic differences between individuals with PTSD compared to healthy controls (Uddin et al., 2010; Smith et al., 2011), particularly in genes involved in inflammation, immune and nervous system function (Uddin et al., 2010; Smith et al., 2011; Rusiecki et al., 2012). Psychosocial stress may alter global and gene-specific DNA methylation patterns potentially associated with peripheral immune dysregulation (Uddin et al., 2010; Smith et al., 2011; Rusiecki et al., 2012).

A larger number of studies have investigated DNA methylation of specific candidate genes, including genes involved in the regulation of the HPA axis (Almli et al., 2014). Epigenetic DNA methylation changes may accompany lifetime experiences and alter gene expression profiles (Yehuda et al., 2015). Significant methylation changes in early life, specifically in genes implicated in developing severe psychiatric conditions including *DLG4, DRD2, NOS1, NRXN1*, and *SOX10* also indicate vulnerability to the effects of stress and psychiatric disorders via epigenetic mechanisms (Numata et al., 2012). Large longitudinal twin and molecular genetic cohort studies suggest that the impact of adverse life events is probably moderated by genetic variants through genetic and environmental interactions (Meaney and Szyf, 2005; Probst et al., 2009).

Post-traumatic stress disorder is associated with the occurrence of multiple comorbidities including depression and coronary heart disease which have been well studied (Sareen, 2014). PTSD is also highly comorbid with chronic pain conditions that often co-occur such as migraine headaches, tension headaches, temporomandibular disorder, irritable bowel syndrome, fibromyalgia, chronic fatigue syndrome and chronic prostatitis/chronic pelvic pain syndrome (Gasperi et al., 2021). Patients with PTSD have a risk of developing pain disorders, which may produce long-lasting changes in the threshold for migraine attacks by inducing epigenetic modifications throughout the brain (Eising et al., 2013). Although several epidemiological studies have reported that PTSD is a predictor of migraine and is much more prevalent in patients with migraine than in the general population (Peterlin et al., 2008; Smitherman et al., 2009; Minen et al., 2016; Zarei et al., 2016), migraine is an understudied comorbidity of PTSD.

Migraine may be aggravated by stress, exercise, sleep deficiency, hormonal changes, head traumas, major depression, PTSD and environmental cues (Theeler et al., 2012; Guglielmetti et al., 2020). In addition, migraine-related pain may cause sensitisation of certain pain pathways via inflammation-induced changes in epigenetic gene regulation (Eising et al., 2013). A recent GWAS reported that migraine showed a higher genetic correlation with psychiatric disorders when compared to other neurological disorders, suggesting common genetic basis or pathways (Anttila et al., 2018). Little is understood about the link between PTSD and migraine disorders, however, it has been suggested that several systems such as the immune system are likely to be involved in the co-occurrence of these disorders (Peterlin et al., 2011). Biological, environmental and genetic risk factors may converge to produce a brain state which predisposes an individual to both PTSD and migraine (Antonaci et al., 2011). Causal pathways shared between migraine and its comorbid disorders may be modulated by epigenetic mechanisms (Eising et al., 2013); these have been suggested to play a role in development of both disorders (McGowan, 2013).

It is important to understand the role genetic, environmental and epigenetic factors play in determining an individual’s susceptibility to PTSD and other co-occurring symptoms (Blacker et al., 2019). In this study we used the disease-discordant monozygotic (MZ) twin design to investigate PTSD-migraine comorbidity. This is a powerful strategy in genetic and epigenetic epidemiology as participants are genetically identical and well-matched by age, sex, maternal environment, population cohort effects and exposure to many shared environmental factors. Recent studies have used this design and uncovered considerable epigenetic (methylation) variation between MZ twins for several complex phenotypic traits which are detectable in blood DNA samples (Bell and Spector, 2011; Bolund et al., 2017; Kaut et al., 2017; Gerring et al., 2018; Peng et al., 2018; Gasperi et al., 2021).

The study aimed to identify PTSD associated genes overlapping with migraine and evaluate similarity in biological pathways between these disorders. To the best of our knowledge this is the first study to use the monozygotic twin design to determine DNA methylation differences between twins discordant for PTSD and migraine.

## Materials and Methods

### Participants and Samples

The study was based on a subset of six pairs of monozygotic (MZ) twins discordant for PTSD and 15 pairs of MZ twins discordant for migraine that were part of a larger cohort of twins recruited by the QIMR Berghofer Medical Research Institute (Wright and Martin, 2004). All twins were of Caucasian descent. Informed written consent was obtained from each participant. All questions were administered using a computer-administered telephone interview (for PTSD). In addition, respondents were requested to complete a brief questionnaire either online or in person which included questions on physical health, personality and other measures (for PTSD and migraine studies). Ethical clearance for this study was obtained through QIMR Berghofer Medical Research Institute along with Queensland University of Technology (QUT) Human Research Ethics Committee approval.

### Assessment of PTSD

The MZ twins discordant for PTSD were part of a larger Missouri Alcoholism Research Center Project 7 (MARC7) study investigating the effects of early experiences and alcohol use in twins, siblings and their spouses in 2010–2013. e-Trauma is a semi-structured interview, which capitalizes on prior research in psychiatric epidemiology and is based on items previously validated by other research interviews, including SCID (Structured Clinical Interview for DSM Disorders) and DSM-IV (Bell, 1994). All twins had experienced a PTSD-qualifying potentially traumatic event as per the DSM-IV criteria. PTSD was assessed via the DSM-IV criteria through structured interview questions that were asked over the phone by an experienced interviewer. PTSD diagnosis was based on the self-reported DSM-IV criteria.

### Assessment of Migraine

The MZ twins discordant for migraine were part of the 25-UP (Mitchell et al., 2019) and Memory attention and problem solving (MAPS) study (Wright and Martin, 2004) at QIMR. Migraine were assessed using the International Headache Society (IHS) diagnostic criteria (the International Classification of Headache Disorders, ICHD-3) together with a diagnosis of migraine with or without aura [Headache Classification Committee of the International Headache Society (IHS), 2013]. For the collection of detailed ICHD-3 diagnostic criteria, participants answering “yes” to ever having “migraine or recurrent attacks of headache” (screening positive), then answered a number of questions relating to their symptoms. Diagnoses were determined for the two major varieties of migraine: migraine without aura and migraine with aura (Launer et al., 1999). MZ twins discordant for migraine with aura were selected for the study.

### DNA Methylation Microarray Analyses

Blood samples were collected from all participants. Purified DNA was quantified on a Qubit Fluorometer (Thermo Fisher Scientific, United States) and for each sample 500 ng was bisulphite-converted using EZ DNA Methylation Kits (Zymo Research, United States). The samples were then assayed for genome-wide DNA methylation levels using Illumina EPIC DNA methylation arrays that offer a high coverage of CpGs >850,000 CpG sites at single-nucleotide resolution, covering all known genes (96% Refseq genes). All procedures were performed according to the manufacturer’s protocol, and arrays were scanned on an Illumina HiScan (Illumina, United States) at the Genomics Research Centre, QUT.

### Statistical Analysis

Raw scan data from the Illumina EPIC arrays were exported into R (V*4.0.2)* for statistical analysis. Samples with probe detection call rates <95% and those with an average intensity value of either <50% of the experiment-wide sample mean or <2000 arbitrary units (AU) were excluded from further analysis. The raw DNA methylation beta values were background and control-normalized using the Bioconductor MINFI package (1.4.0) (Aryee et al., 2014). Cell counts were analyzed using the Houseman method (Houseman et al., 2012). All samples were run in a single batch and were of Caucasian ethnicity. Epigenome-wide differential methylation analysis between case and control groups was performed using linear mixed effects models in R (lmer) to account for the twin pairs and adjusting for cell-counts, age and sex. To correct for multiple testing and identify significant CpGs, we calculated a stringent Bonferroni threshold for significance (*P* = 5.77 × 10^–8^) and a less stringent threshold of 10% false discovery rate (FDR).

The power of a sample was determined using a traditional power calculator based on 50 simulations and expected target delta ranging from 0.20–0.50 at 5% FDR (Graw et al., 2019), with a classical empirical power of 66 and 83% to detect differentially methylated DNA methylation, respectively.

To test whether the overlap of genes between two analyses was more than expected by chance, enrichment testing was performed using 1,000 permutations (using random sets) and applying a two-sided Binominal test in R to give a *p*-value of enrichment.

To assess the biological and molecular mechanisms in PTSD and migraine, pathway and gene set analyses were performed using the KEGG pathway analysis tool via the Webgestalt interface (Wang et al., 2013) to identify enriched pathways using a hypergeometric test for enrichment evaluation analysis and significance level of 10% FDR.

## Results

### Demographics

The study comprized of a total of 21 pairs of MZ twins (*n* = 42). Of these, six pairs of MZ twins were discordant for PTSD while 15 pairs of MZ twins were discordant for migraine. Of the six pairs of MZ twins discordant for PTSD, five pairs were male while one pair was female, with a mean age of 43.3 years [SD = 8.58] across the pairs. Of these MZ twins, 10 of them were male while two were female. Of the 15 pairs of MZ twins discordant for migraine, seven pairs were male while eight pairs were female, with a mean age of 23 years [SD = 9.95] across the pairs. Table 1 shows details of the study participants.

**TABLE 1:** Demographics of the study participants including six pairs of MZ twins discordant for PTSD and 15 pairs of MZ twins discordant for migraine.

Status	Age; mean (SD)	Sex; *n*	
		**Male**	**Female**

**PTSD (*n* = 6)**	43.3	5	1
**Control (*n* = 6)**	43.3	5	1
**Total**	**43.3 (±8.58)**	**10**	**2**
**Migraine (n = 15)**	23.4	7	8
**Control (*n* = 15)**	22.6	7	8
**Total**	**23 ± 9.95**	**14**	**16**

### PTSD Candidate Genes

We investigated 60 PTSD candidate genes that had been previously reported to be associated with PTSD in different studies (Cornelis et al., 2010; Almli et al., 2014; Mehta et al., 2019; Bian et al., 2020; Stein et al., 2021), including genes uncovered in a GWAS of over 250,000 participants in the Million Veteran Program (Stein et al., 2021). For these 60 candidate genes, there were 4411 CpGs detected in the current study on the EPIC microarray that could be tested. We tested for association between DNA methylation and PTSD status in the twin pairs using linear mixed effects (lmer) models with age, sex and cell counts as covariates.

**FIGURE 1:**
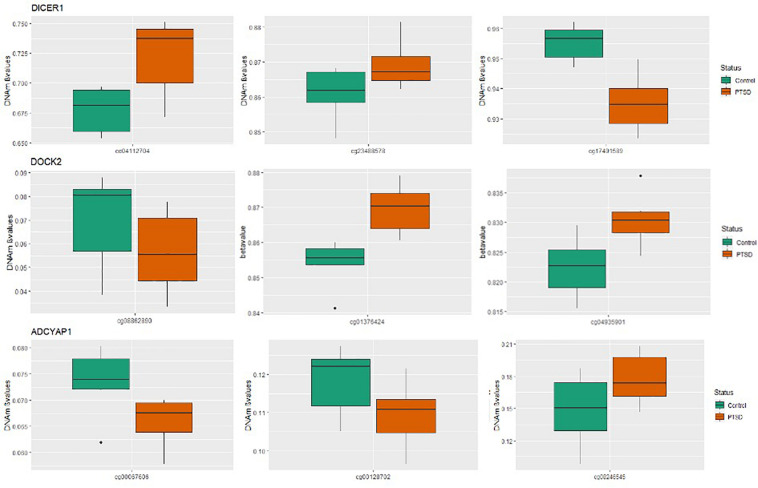
Boxplots for significant PTSD candidate genes: Box plots of DNA methylation level beta-values at significant candidate genes for PTSD versus non-PTSD (control) MZ twins that were significant at gene-wise Bonferroni correction. The mean DNA methylation is higher in PTSD than in controls for two of the three CpGs for DICER and DOCK2, while ADCYAP1 has only one CpG with a higher mean DNA methylation in individuals with PTSD compared to control twins. The remainder of the CpGs have a higher mean DNA methylation in controls than with PTSD.

Of the 4411 CpGs tested, 440 CpGs from 54 genes were significantly associated with PTSD (*p* < 0.05). There were 14 genes with at least one CpG surviving the locus-specific Bonferroni correction, including *DOCK2, SLC6A3, DICER1, DRD2, ADCYAP1, ADCYAP1R1, SKA2, OXTR, STMN1, SLC6A4, DBH, ZNF626, TRAIP, TSNAIRE1*, and *IMMP2L.* Details of the top candidate genes including the number of CpGs significant within each gene are shown in Table 2. Based on 1,000 permutations, this indicates significant overlap above what would be expected by chance (enrichment *p*-value = 0.0098). Full results are shown in Supplementary Table 1. Figure 1 illustrates DNA methylation differences between PTSD and non-PTSD for three of the top candidate genes.

**TABLE 2:** PTSD Candidate genes with at least one CpG significant at gene-wise Bonferroni threshold for significance.

Gene symbol	Number of CpGs tested	≧1 CpG with *p* ≦ 0.05 (No of CpGs with *p* ≦ 0.05)	Survive bonferroni
DOCK2	96	YES (6)	YES
SLC6A3	81	YES (8)	YES
DICER1	49	YES (5)	YES
DRD2	41	YES (4)	YES
ADCYAP1	40	YES (3)	YES
STMN1	38	YES (1)	YES
ADCYAP1R1	36	YES (6)	YES
SLC6A4	31	YES (2)	YES
SKA2	25	YES (4)	YES
OXTR	22	YES (3)	YES
DBH	18	YES (3)	YES
ZNF626	9	YES (1)	YES
TRAIP	22	YES (2)	YES
TSNARE1	215	YES (27)	YES
IMMP2L	96	YES (10)	YES

### Genome Wide Analysis for PTSD Status and DNA Methylation

Next, we performed a hypothesis-free epigenome-wide association analysis to test for DNA methylation differences between the MZ twins discordant for PTSD across all 866K CpG sites on the array. Association testing was performed using lmer models and adjusting for cell counts, age and sex in the model as possible confounders.

**FIGURE 2:**
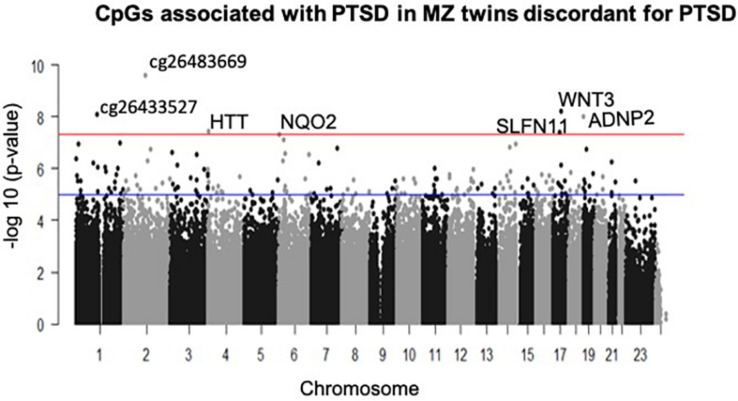
Manhattan plot of CpGs associated with PTSD: the blue line indicates Bonferroni threshold of significance (<5.77 × 10^– 8^) and the red line indicates 10% FDR. A total of 7 CpGs were significant at Bonferroni threshold and 1,585 CpGs were significant at 10% FDR.

**FIGURE 3:**
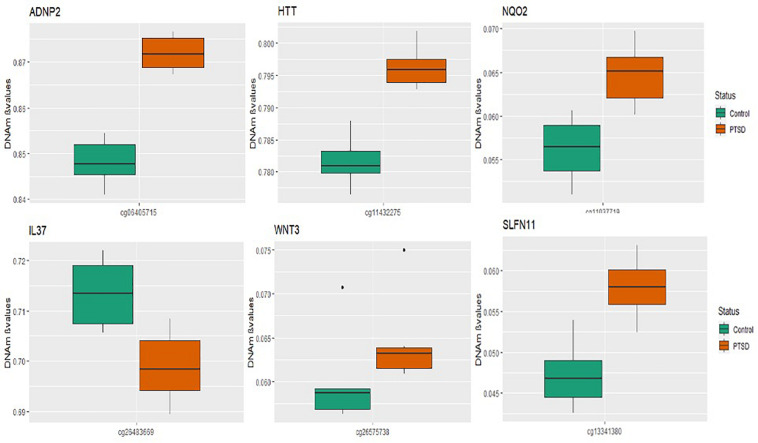
Box plots of most significant genes: boxplots of genes significantly associated with PTSD (*p*-value < 5.77 × 10^– 8^) in the MZ twins is depicted. The mean DNA methylation is higher in PTSD than in controls for all the genome wide significant genes except for the IL37 locus probe.

A total of seven CpGs were significant at the Bonferroni level of significance of *p* < 5.77 × 10^–8^ (Table 3). Full results across all the CpG sites are shown in Figure 2 and Supplementary Table 2, a total of 159 CpGs (115 genes) were significant at 5% FDR. The strongest association was detected for chromosomes 1, 2, 4, 6, 17, and 18. The top CpGs were included *IL37 cg26483669, CSF1 cg26433527, ADNP2 cg06405715, WNT3 cg26575738, NQO2 cg11037719, HTT cg11432275*, and *SLFN11 cg13341380* (Figure 3). The CpG in *IL37* showed hypomethylation in the PTSD twins while the other CpGs in *CSF1, ADNP2, WNT3, NQO2, HTT*, and *SLFN11* were hypermethylated in PTSD. As DNA methylation signals across neighboring CpGs can be highly correlated, we used a less conservative threshold of significance (10% FDR) to identify other genes of interest in PTSD. This included 1453 CpG sites in 1036 genes that were significant at 10% FDR. Several of these genes such as *HDAC4* and *NRG1* were known to be affected by a known drug (*n* = 84 genes, clinically actionable^∗∗^) or had genomes that could be used to build new drugs (*n* = 248 genes, druggable genomes^∗^) as per the Drug Gene Interaction Database (Cotto et al., 2017; Supplementary Table 2).

**TABLE 3:** List of CpG sites significantly associated with PTSD at Bonferroni threshold for significance (*p* < 5.77 × 10^–8^).

Cpg	*P*-value	Chromosome	Basepair^a^	Gene symbol (closest gene)	CpG location to gene	Direction PTSD
cg26483669	2.47E-10	2	113717037	IL37	Intergenic	Downregulated
cg06405715	9.90E-09	18	77895056	ADNP2	Gene body	Upregulated
cg26433527	8.45E-09	1	110370709	CSF1	Intergenic	Upregulated
cg26575738	6.11E-09	17	44896168	WNT3	TSS200	Upregulated
cgl 1037719	4.72E-08	6	2999695	NQ02	TSS1500	Upregulated
cgl 1432275	3.46E-08	4	3239323	HTT	Gene body	Upregulated
cgl3341380	4.15E-08	17	33701529	SLFN11	TSS 1500	Upregulated

*^*a*^Position in Human GRCh37/hg19 Assembly. TSS, distance from transcription start site.*

### Overlapping Genes and Pathways in PTSD and Migraine

Posttraumatic stress disorder often occurs with other comorbidities, including migraine. As little is known about the basis for this comorbidity, we aimed to assess the occurrence of DNA methylation variation on PTSD candidate genes in association with migraine. Using the same study design as the PTSD sample, we investigated 15 pairs of MZ twins discordant for migraine. We tested for association of methylation at genetic loci that overlap between PTSD and migraine using several different analyses.

#### Known PTSD Candidate Genes Associated With Migraine in the MZ Twins

First, we tested 2,569 CpGs for the 60 PTSD candidate genes identified from earlier studies among the twins with migraine. There were 368 CpGs across 51 genes that had at least one significant CpG associated with migraine (*P* < 0.05). Of these genes, 11 genes survived locus specific Bonferroni corrections for multiple testing including *ADCYAP1, AIM2, CRHR1, DBH, DOCK2, FKBP5, HTR3A, OXTR, RORA, WWC1*, and *TSNAIRE1* (Table 4 and Supplementary Table 3).

**TABLE 4:** PTSD Candidate genes also associated with migraine in MZ twins with at least one Bonferroni significant CpG site.

Gene symbol	Number of CpGs tested	≧1 CpG with *p* ≦ 0.05 (No of CpGs)	Survive bonferroni
ADCYAP1	40	YES (4)	YES
AIM2	16	YES (1)	YES
CRHR1	70	YES (7)	YES
DBH	18	YES (2)	YES
DOCK2	96	YES (8)	YES
FKBP5	53	YES (9)	YES
HTR3A	20	YES (4)	YES
OXTR	22	YES (3)	YES
RORA	238	YES (21)	YES
WWC1	77	YES (5)	YES
TSNARE1	215	YES (13)	YES

#### Epigenome-Wide Overlap of Genes Associated With PTSD Also Associated With Migraine in the MZ Twins

**FIGURE 4:**
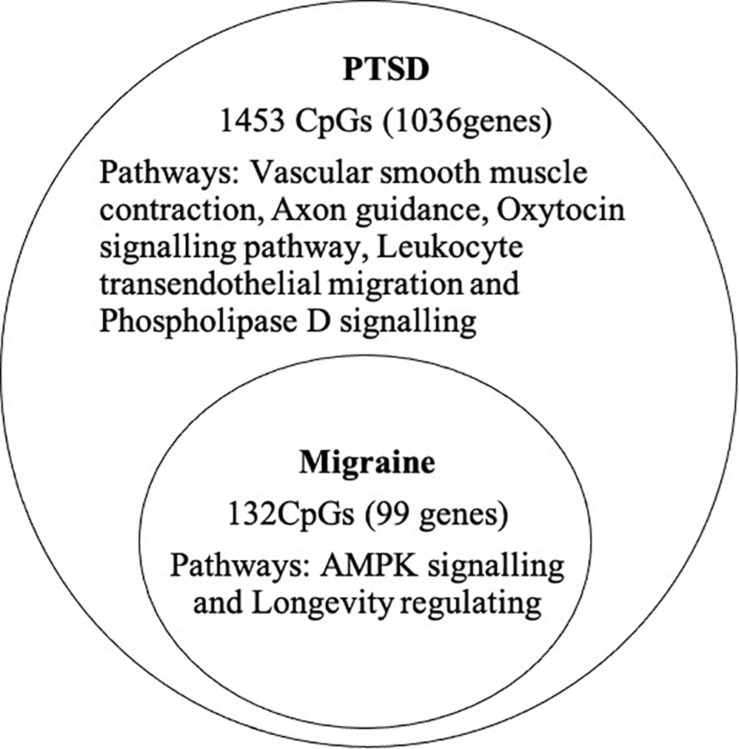
Overlap of PTSD and migraine: Venn diagram of CpGs and genes significantly associated with PTSD (10% FDR) and also associated with migraine (*p* < 0.05).

**FIGURE 5:**
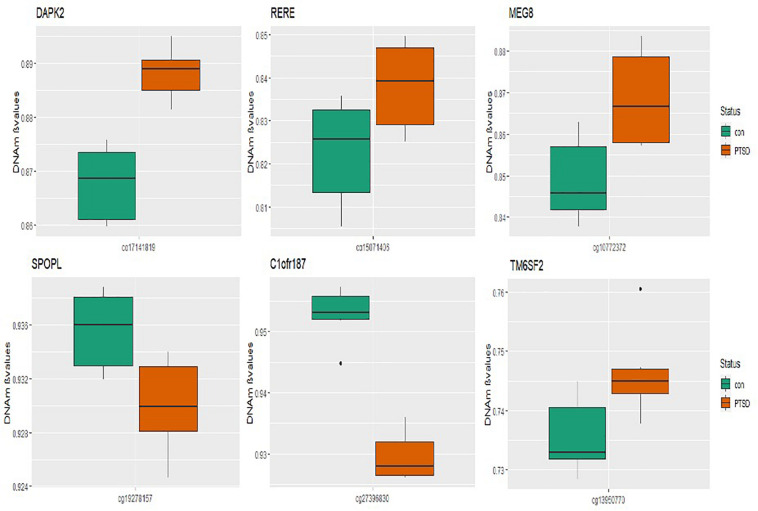
Boxplots of overlapping genes: box plots of PTSD associated genes overlapping in migraine. The mean DNA methylation is higher in PTSD than in controls for all the overlapping genes in migraine except for *SPOPL* and *C1orf187.*

At the epigenome-wide level, we assessed how many of the 1036 genes (1,453 CpGs) associated with PTSD at 5 and 10% FDR overlapped with those significantly associated with migraine in the discordant migraine MZ twins. At 5% FDR, DNA methylation of 13 CpGs (six genes) and at 10% FDR DNA methylation of 99 genes (132 CpGs loci) associated with PTSD was also associated with migraine (*p* < 0.05, Figure 4). At 10% FDR, 47 CpGs were down-regulated (hypo-methylated) in PTSD of which 25 were also downregulated in migraine while 85 CpGs were up-regulated (hypermethylated) in PTSD of which 53 were also up-regulated in migraine (Supplementary Table 4). The overlapping genes included RERE, MEG8, SPOPL, C1orf187 (DRAXIN), DAPK2, and TM6SF2 (Figure 5).

#### Genes Previously Associated With Migraine DNA Methylation

To the best of our knowledge there has only been one study so far investigating comprehensive epigenome-wide DNA methylation changes in migraine performed by Gerring et al., 2018. The study had identified a total of 62 genes associated with migraine (Gerring et al., 2018). Using these genes (62 genes across 2,351 CpGs) we tested how many CpGs within these genes were also associated with migraine and PTSD using the MZ twin sample.

In the migraine MZ twins, for 46 genes out of 62, there was at least one CpG (*p* < 0.05) significantly associated with migraine in the current study. Of these, for 6 genes (*KCNG2, DGKG, SND1, LHX6, ADIRF*, and *RPTOR*), the results survived multiple testing correction at 10% FDR.

In the PTSD MZ twins, we identified that 216 out of 2351 tested CpGs (4 genes i.e., *RPTOR, NUFIP1, SLC38A4*, and *KCP*) were also significantly associated with PTSD (10% FDR) in the current study. Based on 1000 permutations, this overlap is significantly higher than expected by chance alone (enrichment *p*-value = 0.036), providing support for overlapping genes between migraine and PTSD.

#### Functional Annotation of Overlapping Genes

Pathway analysis was performed to determine the biological and molecular function associated with PTSD only and both migraine and PTSD using the KEGG pathway through an online interface (Liao et al., 2019). For genes associated only with PTSD, vascular smooth muscle, axon guidance and oxytocin signaling pathways were overrepresented, all these are well-known pathways for PTSD. For genes associated with both PTSD and migraine, the overrepresented pathways were AMPK signaling (Enrichment Ratio = 9.73, *p*-value = 1.40E-04) and longevity regulating (Enrichment Ratio = 10.49; *p*-value = 5.25E-04). Detailed results are shown in Table 5.

**TABLE 5:** Biological pathways overrepresented among genes associated among genes associated with PTSD (only) and genes associated with both PTSD and migraine.

PTSD Migraine overlap					
Pathway	Number of genes	Enrichment ratio	*P*-value	FDR	Genes within pathway
AMPK signaling pathway	5	9.7252	1.40E-04	0.0433	AKT3; CREB5; IRS1; MLYCD; TSC2
Longevity regulating pathway	4	10.4901	5.25E-04	0.0811	AKT3; CREB5; IRS1; TSC2
**PTSD only**					
Vascular smooth muscle contraction	17	3.4978	5.86E-06	0.0017	ACTA2; ADCY2; ADCY9; ARHGEF12; CACNA1C; CACNA1S; CALD1; GNA13; KCNMA1; MRVI1; MYH11; MYL6B; MYLK2; MYLK3; NPR2; PLA2G6; PPP1R12A
Axon guidance	19	2.7030	7.05E-05	0.0107	ABL1; ABLIM2; ARHGEF12; CAMK2B; CXCL12; EFNA5; ENAH; EPHA4; EPHA5; NGEF; NTN1; PAK6; PARD3; PLCG1; PLCG2; PPP3CB; PRKCZ; ROBO2; TRPC1
Oxytocin signaling pathway	17	2.7844	1.18E-04	0.0119	ADCY2; ADCY9; CACNA1C; CACNA1S; CACNB2; CAMK1D; CAMK2B; KCNJ5; CACNA2D3; MAP2K5; MYL6B; MYLK2; MYLK3; NPR2; PPP1R12A; PPP3CB; RYR3
Leukocyte transendothelial migration	13	2.8897	5.18E-04	0.0396	ACTN1; CLDN1; CTNNA1; CTNNA2; CTNND1; CXCL12; JAM2; MYL12B; PLCG1; PLCG2; RAPGEF4; SIPA1; THY1
Phospholipase D signaling pathway	15	2.5578	7.34E-04	0.0449	ADCY2; ADCY9; AGPAT1; AGPAT3; AGPAT4; ARF1; DGKH; GNA13; PDGFC; PLCG1; PLCG2; RALB; RAPGEF4; SHC1; TSC2

## Discussion

PTSD often co-occurs with other disorders and migraine being one of the less-studied comorbidities of PTSD. In the current study, we analyzed epigenome-wide DNA methylation data in MZ twins discordant for PTSD and migraine to identify common genes and pathways in PTSD and migraine. DNA methylation can be affected by both genetic and environmental factors. Therefore, the MZ twin’s disease-discordant design is a powerful approach to dissect overlap between disorders as the participants are genetically and demographically matched (Bell and Spector, 2011; Tan et al., 2015).

Using this unique study design, we first investigated six pairs of MZ twins that were all exposed to stress but were discordant for PTSD. Genome-wide analyses of DNA methylation differences across the twins identified 7 CpG loci that were significantly associated with PTSD even after stringent Bonferroni correction for multiple testing. The top CpG was in an intergenic region on chromosome 2, located near gene *IL37*. This gene has not been directly implicated in PTSD and is an anti-inflammatory cytokine that has been found to be increased in the amygdala and dorsolateral prefrontal cortex of children with autism spectrum disorder (Tsilioni et al., 2019). The cg26433527 site is upstream of the *CSF1* gene, a cytokine that controls the production, differentiation, and function of macrophages.

Other genome-wide significant genes included *WNT3, ADNP2, SLFN11* and *HTT. WNT3* has been associated with stress-induced depression-like behaviors (Zhou et al., 2016) while *ADNP2* has been suggested to cause changes in cellular viability under oxidative stress (Kushnir et al., 2008). *SLFN11* acts as a global regulator of chromatin structure with the potential to engage the innate immune activation in response to replicative stress (Murai et al., 2020). The *HTT* is the Huntingtin gene involved in Huntington’s disease, a neurodegenerative disorder characterized by loss of striatal neurons. This is in line with our previous finding of an involvement of *DOCK2* in PTSD, a gene which has also been implicated in the formation of amyloid plaques in the brain in Alzheimer’s disease (Mehta et al., 2017), suggesting the role of genes common to both PTSD and neurodegenerative disorders.

When assessing 45 candidate genes known to be involved in PTSD through other studies, we found that DNA methylation of these genes were significantly more likely to be associated with PTSD in the current study than expected by random chance. Of the PTSD candidate genes, we found 11 genes were also associated with PTSD in the current twin study after gene-wise Bonferroni correction (see Table 2). The candidate genes included *DOCK2, SLC6A3, DICER1, DRD2, ADCYAP1, ADCYAP1R1, SKA2, OXTR, STMN1, SLC6A4, DBH, ZNF626, TRAIP, TSNAIRE1*, and *IMMP2L.* Our team has previously found *DOCK2*, an amyloid-plaque associated gene in Alzheimer’s, to be associated with PTSD in an epigenome-wide study in Australian veterans (Mehta et al., 2017) and here have validated this gene in the MZ PTSD twins. Another PTSD candidate gene *SLC6A3* encodes the dopamine transporter, individuals with PTSD carrying the *SLC6A3* 9-repeat allele were found to be at higher risk for PTSD when also having higher methylation in the *SLC6A3* promoter locus (Chang et al., 2012). SLC6A4 encodes the serotonin transporter. A study reported increased methylation of *SLC6A4* in bullied twins at age 10 compared to their non-bullied monozygotic twins (Ouellet-Morin et al., 2013). In genome wide studies, reduced *SLC6A4* methylation levels were associated with more traumatic events and increased risk for PTSD in individuals carrying a specific *SLC6A4* risk allele genotype while higher *SLC6A4* methylation appeared protective against the development of PTSD (Koenen et al., 2011). The contrary results of *SLC6A4* are likely driven by a gene-by-environment interaction whereby the particular genotype interacts with environment (trauma) to induce DNA methylation changes and confer susceptibility towards PTSD. Other stress-related candidate genes include *SKA2* and *ADCYAP1R1; a* study showed associations between the methylation status, a polymorphic site in the 3′UTR of the *SKA2* gene—involved in mitosis—with reduced thickness of several cortical areas and symptom severity in PTSD (Daskalakis and Yehuda, 2014). Methylation in peripheral blood samples of ADCYAP1 (PACAP protein) and its receptor *ADCYAP1R1* (PAC1 protein), genes involved in regulating the cellular stress response, were associated with PTSD diagnosis and symptom severity, specifically in females (Ressler et al., 2011). There is emerging evidence of a link between PACAP and migraine, with clinical trials targeting PACAP as a likely prevention for migraine (Sundrum and Walker, 2018; Rustichelli et al., 2020).

Posttraumatic stress disorder and chronic pain conditions often occur together, and the underlying mechanisms are diverse and multifactorial (Smitherman et al., 2009; Minen et al., 2016; Gasperi et al., 2021). To investigate genes involved in both PTSD and migraine, we compared genes associated with PTSD in the current study and tested whether the same genes were also associated with migraine in an independent sample of 15 MZ twins discordant for migraine. We identified *DAPK2* and *TM6SF2* as two of the top overlapping genes between the two disorders. *DAPK2* is a calmodulin-regulated protein kinase, it has been implicated in the intracellular degradation process essential for adaptation to metabolic stress (autophagy) (Shiloh et al., 2018). *TM6SF2* is associated with cardiovascular disease and plays a role in oxidative stress (Taliento et al., 2019). These findings suggest that epigenetic changes in response to different types of stress may “mediate” stress phenotypes. Stress is a possible underlying cause of both PTSD and migraine and can impact the epigenome (Fuchikami et al., 2009; Schouten et al., 2013; Chakravarty et al., 2014; Kenworthy et al., 2014) and damage the brain in severe cases (McEwen, 2006, 2013). For instance, a single immobilization stress alters hippocampal brain-derived neurotrophic factor (*BDNF*) gene expression and histone acetylation at *BDNF* gene promoters (Fuchikami et al., 2009). *BDNF* is involved in the neural plasticity underlying the extinction of fear (Chhatwal et al., 2006; Heldt et al., 2007; Soliman et al., 2010) and recovery from stress. The *BDNF g*ene has been studied in relation to anxiety disorders such as PTSD (Ressler et al., 2011; Andero and Ressler, 2012). Increased methylation of a CpG site in *BDNF* and peripheral blood of adults with PTSD have been reported (Smith et al., 2011). Studies have shown that *BDNF* is increased during migraine attacks, and in cluster headache (Fischer et al., 2012), and there is some genetic evidence suggesting a role of *BDNF* in migraine (Sutherland et al., 2014).

Another well-studied PTSD candidate is Catechol-O-methyltransferase (*COMT*), which encodes enzymes that degrade neurotransmitters such as dopamine and the serotonin transporter *SERT*. Increased *COMT* promoter methylation was associated with impaired fear inhibition in individuals with PTSD carrying the *COMT* met/met genotype (Meyer-Lindenberg and Weinberger, 2006). Polymorphisms in the *BDNF*, *COMT*, and *SERT* lead to disturbances in the normal brain pathway neurotransmitters making patients more susceptible to several neuropsychiatric disorders (Andersen and Skorpen, 2009; Fischer and Jan, 2020). Interestingly, *COMT* has been associated with headache medication overuse (Fischer and Jan, 2020). These studies further supporting the involvement of similar stress-related genes in the pathophysiology of both PTSD and migraine. Other proposed neurobiological mechanisms underlying PTSD-migraine comorbidity includes dysfunction of the autonomic nervous system, Hypothalamic Pituitary Adrenal (HPA) axis and brain serotonergic dysfunction (Smitherman et al., 2009; Minen et al., 2016).

When assessing biological pathways implicated in genes associated with PTSD and migraine, we found that several different pathways were involved in genes implicated in PTSD only versus those implicated in PTSD and migraine. For instance, vascular smooth muscle, axon guidance and oxytocin signaling pathways were overrepresented for genes associated with PTSD; all these are plausible and well-known pathways for PTSD. Interestingly, migraine GWAS studies have also identified an abundance of vascular genes associated with migraine (Mason and Russo, 2018; van den Maagdenberg et al., 2019; Guo et al., 2020). For genes associated with both migraine and PTSD, AMPK signaling and longevity regulating pathways were overrepresented. Adenosine monophosphate-activated protein kinase (AMPK) pathway activation might be a therapeutic target for PTSD (Wang et al., 2017) and AMPK activators might also be effective for treatment of chronic pain disorders by inhibiting signaling pathways that promote changes in the function of peripheral nociceptive neurons (Asiedu et al., 2016). There is not much known about the role of longevity pathways in PTSD or migraine.

Epigenetic regulation of gene expression is a dynamic and reversible process and therefore a good pathway target with drugs (Eising et al., 2013). In our study we used the Drug Gene Interaction Database (DGIdb) for all genes associated with PTSD to check if they were known to be affected by a known drug (clinically actionable) or are genes or encode gene products that are known or predicted to be targets for new drugs (i.e., a druggable genome). Of genes significantly associated with PTSD at 10% FDR, we found that 7.4% of these genes were known and clinically actionable targets for drugs while a further 22% of genes had a druggable genome. It has been suggested that defining the exact nature of this association and the pathophysiological mechanisms underlying the comorbidity are relevant in clinical practice as it might influence both the response to treatment and likelihood to achieve remission (Dresler et al., 2019; Seng and Seng, 2016). Current acute and prophylactic treatments are effective in less than half of the patients (Goadsby et al., 2002), indicating the need for more effective drugs. There are currently no epigenetic drugs that have gone into clinical trials for the treatment of neuropsychiatric disorders or migraine with the exception of valproate (Peedicayil and Kumar, 2012; Eising et al., 2013). Identifying factors that predispose to migraine attacks is therefore crucial to provide specific molecular targets to design novel migraine drugs. Understanding which genes are involved in co-morbid disorders such as PTSD and migraine will support the development of more advanced psychological and/or pharmacological interventions that target key pathways underlying both disorders.

There are several limitations of this study including small sample size (42 samples) which has limited power to detect small DNA methylation changes. Given the unique study design of genetically and demographically matched samples, this is a small yet homogenous sample to detect changes in disease. Another limitation of the study is the use of peripheral blood to assess DNA methylation in PTSD and migraine and while this has been a topic for discussion for many years, it is now evident that whole blood acts as a relevant and easily accessible surrogate to investigate brain-disorders. We also used an online tool by Hannon et al. to investigate the correlation of DNA methylation in blood for the significant genes with four brain regions (Ligthart et al., 2016). The modest overlap in genes and pathways between PTSD and migraine might be due to several factors including limited power and differences across the two MZ twin samples compared. Given the cross-sectional nature of the DNA methylation measurements, it is difficult to disentangle cause from effect. It is recommended that future studies should use large cohorts and at various time points to identify biological underpinnings of PTSD and migraine.

Nevertheless, we were able to validate and replicate several previously reported findings from PTSD in this study and this is the first study of its kind to use a MZ-twin design to disentangle the genes overlapping in PTSD and migraine. Using the disease discordant MZ twin design is a powerful approach in EWAS as the participants are genetically matched with similar environmental exposure and lifestyle especially in the earlier years (Tan et al., 2015). Twin studies provide a useful reference for hypotheses to be replicated and validated to understand epigenetics of complex diseases (Bell and Spector, 2011).

There are several proposed mechanisms underlying the psychiatric comorbidities and migraine relationship, but their exact etiology and biological mechanisms are not entirely known (Smitherman et al., 2009; Eising et al., 2013). Although there have been promising developments in genome-wide studies on psychopathologies in recent years, data on the biological basis of PTSD and migraine are limited. Few studies with small populations have been conducted to examine genetic and epigenetic (methylation) associations in PTSD (Uddin et al., 2010; Mehta et al., 2018). There is need for comprehensive evaluation and integrated model of care of psychiatric disorders in migraine (Dresler et al., 2019). More research is required to identify epigenetic targets that affect migraine pathophysiology and drugs that specifically act to modulate chromatin structure at migraine pathways (Eising et al., 2013; Minen et al., 2016).

To the best of our knowledge, this is the first study to examine overlapping genes and biological processes in PTSD and migraine comorbidity using the monozygotic co-twin design. These results are important and suggest that common genes and pathways might be associated with PTSD and migraine, with implications for diagnosis of comorbidities in PTSD and common treatments for these co-morbid disorders.

## Data Availability Statement

The datasets presented in this study can be found in online repositories. The names of the repository and accession number can be found below: Gene Expression Omnibus GSE172464.

## Ethics Statement

The studies involving human participants were reviewed and approved by QIMR Berghofer Medical Research Institute and Queensland University of Technology (QUT) Human Research Ethics Committee. The patients/participants provided their written informed consent to participate in this study.

## Author Contributions

CB analyzed the data and wrote the first draft of the manuscript with DM support and guidance. NM, EN, AH, IH, and MW led studies and provided data from QIMR Berghofer Medical Research Institute. HS and RP conducted the laboratory sample analysis. LH and LG contributed to head of the Genomics Research Centre at QUT and supervised all experimental protocols. AM provided statistical advice and input. DM and DN conceived the study and supervised the project. All authors approved the manuscript and provided critical comments on the manuscript.

## Conflict of Interest

The authors declare that the research was conducted in the absence of any commercial or financial relationships that could be construed as a potential conflict of interest.

## Supplementary Material

Supplementary Figure 1 Correlation of DNA methylation in blood and brain for MEG8 gene.

Supplementary Table 1 A list of all PTSD Candidate genes tested in the MZ twins.

Supplementary Table 2 List of genes significantly associated with PTSD (10% FDR) and overlapping with migraine in the MZ twins.

Supplementary Table 3 A list of all PTSD Candidate genes associated with migraine in the MZ migraine twins.
