# Supplementary figures and images for: Using Monozygotic Twins to Dissect Common Genes in Posttraumatic Stress Disorder and Migraine
Source: Front Neurosci. 2021 Jun 22;15:678350. doi: 10.3389/fnins.2021.678350 (PMC8258453; doi:10.3389/fnins.2021.678350)

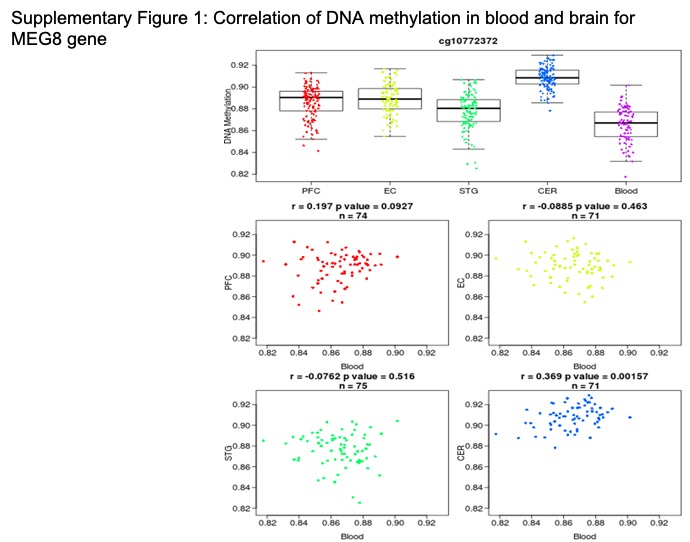

Supplement: Supplementary Figure 1 — Correlation of DNA methylation in blood and brain for MEG8 gene. [file Image_1.JPEG]
